# Supplementary material for: Assessing the severity of laparotomy and partial hepatectomy in male rats—A multimodal approach
Source: PLoS One. 2021 Aug 2;16(8):e0255175. doi: 10.1371/journal.pone.0255175 (PMC8328343; doi:10.1371/journal.pone.0255175)
Supplement: S1 File — Experiment-specific score sheet for daily severity assessment according to Morton DB et al. (PDF) [file pone.0255175.s001.pdf]

Animal ID:	Score:	POD 1	POD 2	POD 3	POD 4	POD 5	POD 6	POD 7
**Date**								
**I body weight:**								
unaffected or increase	0							
decrease <5 %	1							
decrease <10%	5							
decrease <19%	10							
decrease ≥ 20%	20							
body weight	g:							
**II general condition:**								
fur smooth, shiny; body orifices clean; eyes clear, shiny; unimpaired appearance	0							
minor fur defects, slightly disordered fur, unkempt coat around eyes (red tears, eye ointment residues, etc.)	1							
dull or disordered, shaggy fur; unkempt body opening; eyes cloudy, slightly closed; slightly altered muscle tone (staggering, writhing); frequent stretching (back-arching, etc.)	5							
dirty fur, abnormal posture (raised back, pain face); high muscle tone; dehydration; altered breathing; diarrhoea (<48 h)	10							
cramps; paralysis; difficulty breathing; icterus; diarrhoea (>48h); animal feels cold; permanent crouching with closed eyes	20							
**III spontaneous behaviour and willingness to move:**								
normal behaviour (sleeping, reaction to touch, curiosity, raising the edge of the cage, social contacts, social interaction, cleaning)	0							
minor deviations from normal behaviour; protective posture of the operated limb during rearing	1							
unusual behaviour; motor abnormalities (stilt gait, protective posture of the operated limb in the gait); reduced willingness to move (only after impulsion)	5							
isolation; apathy; strongly reduced willingness to walk; behavioural stereotypes/aggressiveness; coordination disorders	10							
pain noise during grabbing; lethargy; no readiness to move; automutilation	20							
**IV surgery-associated and trial-specific parameters**								
wound healing unimpaired, no swelling	0							
wound with minor redness, suture ends gnawed off (wound closed)	1							
Wound redness, minor wound swelling, slight manipulation of the wound (wound closed)	5							
wound secretion, gnawing on wound, wound infection, suture dehiscence (discontinuous wound margins), minor postsurgical bleeding, swelling of tongue	10							
Repeated suture dehiscence, severely infected wound, haemascosis, high-grade swelling of the tongue with opened mouth, impaired breathing	20							
**Assessment, actions:**								
assessment level 0: no severity	0							
assessment level 1: mild severity, continue to monitor carefully	1-9							
assessment level 2: moderate severity; if necessary, initiate veterinary care (analgesia, in case of suture dehiscence: renewed wound care/closure under anaesthesia).	10-19							
assessment level 3: severe severity (humane endpoint); immediate termination of the experiment, euthanise animal	>20							
