# Supplementary material for: Assessing the severity of laparotomy and partial hepatectomy in male rats—A multimodal approach
Source: PLoS One. 2021 Aug 2;16(8):e0255175. doi: 10.1371/journal.pone.0255175 (PMC8328343; doi:10.1371/journal.pone.0255175)
Supplement: Attachment — Submitted filename: Response to Reviewers_PONE-D-21-06410R1.pdf [file pone.0255175.s004.pdf]

Response letter PONE-D-21-06410

Dear Madam/Sir:

First of all, we would like to thank the Editors and the Reviewers again for their valuable input to further improve our manuscript.

Below, you will find our response to the individual comments of the 1st reviewer as a point-to-point response letter.

Review Comments to Author:

1. **Thanks for including the severity score sheet. This is very helpful for the reader. We would recommend to refer to the score sheet in the supplementary material in the method section on severity scoring. Otherwise this sheet might be overlooked.**

   Thank you very much for this important recommendation. We added a note, to refer to the supplementary materials, in lines 203 – 205 of the revised manuscript.

   "For severity scoring, a modified version of the scoring system described by Morton et al. (1985) [12] was used daily to assess the general condition of rats (see complete score sheet in supplementary material)."

2. **Line 209 Additional surgery in animals with wound closure problems: Thanks for describing the procedure of re-closure of surgical wounds. We wonder how you handled the data gathered from the animal that was subjected to an additional anesthesia? Were they included in the results? The authors should state whether they think the second surgery is influencing there results and if this animal should be possibly excluded from the study. Maybe a short sentence could be added on the subject.**

   Yes, data of re-sutured animals were included in the analysis. We think, that wound healing disorders and surgery-related complications are a physiological bias to a certain extent, which should not be excluded. Furthermore, these wound healing disorders were pre-defined in the score sheet and do not constitute a reason for the exclusion of animals from both, animal welfare and a research perspective. In addition, biological variability of about 20% was already considered in the design of the experiment. In conclusion, even under the impact of these data (9 out of 136 included animals, ≤ 7%), the severity grades were evaluated as low and less severe than assessed by the EU-Directive.

   Please see lines 212 – 219 of the revised Material and Methods section:

   "To assess wound healing, several stages with subsequent proceedings were defined. If only the thread ends were gnawed off (wound edges adapted), a more intensive monitoring was performed. If the adaptation of wound edges was about to loosen, a wound clip was applied on the corresponding area to secure the suture, and was removed as soon as the wound looked resilient. If a suture dehiscence of single knots occured, the animal was re-anesthetized, the wound was cleaned, and re-sutured. In the case that the suture of the abdominal muscle layer ruptured (hernia; skin suture closed), the animal was re-anesthetized, skin sutures were removed and finaly muscles layer and skin were re-sutured."

Further, in lines 295 - 297:

"Data of re-sutured animals or animals with surgery-related complications were included in the analysis, as long as they did not reach the predefined humane endpoints."

And lines 325 – 329 of the revised Results:

"Further wound healing disorders occurred in the post-surgical recovery period: two skin sutures had to be fixed with additive wound clips (PH70%); one skin suture had to be re-sutured under short general anesthesia (SHAM group); six animals got a hernia of the muscle layer and had to be re-sutured under general anesthesia (PH70%). Overall, the scores …"

To make this approach transparent to the reader, we added the following text to the discussion. Please see lines 519 – 522:

"Although, data of re-sutured animals were included in the analysis, we think that wound healing disorders and surgery-related complications are a physiological bias to a certain extent, which should not be excluded. Even under the impact of these data (9 out of 136 included animals, ≤ 7%), the severity grades were evaluated as mild to moderate."

3. **Blood parameters: the authors gave a satisfying answer to our question why blood parameters were applied. This should be included in the manuscript.**

   We thank you for this improving hint and hope, that the additional information points out why blood parameters were assessed at all. Therefore, we included the sentences in the results and the discussion section of the manuscript.

   Please see lines 407 - 409 of the revised manuscript:

   "… Metamizole is known to cause agranulocytosis in patients [18], which we could not confirm in our animals. To identify and verify metamizole-dependent changes, which could have an impact on severity grades, the blood cell counts were assessed. …"

   And line 505 – 507:

   "While surgical interventions can lead to inflammation and thus to changes in the white blood cell counts, bleeding that may be caused by liver resection, can lead to changes in the red blood cell counts."

4. **Metamizol treatment Line 203ff: Please check the Metamizole dosage in the revised manuscript. Was the s.c. dosage equal to the dosage in the drinking water?**

   We apologize for the misleading wording. We added detailed information to each application time point to clarify the different treatments and dosages. Please see lines 191 – 196:

   "Analgesia was administered subcutaneously (metamizole, Novaminsulfon-ratiopharm® 1 g/2 mL; 100 mg/kg, single dose) prior to each surgery. For post-surgical pain management, metamizole (Novaminsulfon-ratiopharm® 1 g/2 mL; 400 mg/kg/day, oral) was administered until the late afternoon of the third day after surgery (POD 3), via sweetened drinking water."

We hope that our answers meet the reviewers´ expectations and that the revised version is now acceptable for publication in your esteemed journal.

With best regards,

Sincerely,

Rene H. Tolba
Corresponding author
